# A multicenter study comparing the outcome of catheter ablation of atrial fibrillation between cryoballoon and radiofrequency ablation in patients with heart failure (CRABL‐HF): Study design

**DOI:** 10.1002/joa3.12322

**Published:** 2020-03-15

**Authors:** Koji Miyamoto, Kanae Hasegawa, Hiroki Takahashi, Yoh Masue, Naoya Kataoka, Koji Fukuzawa, Keiichi Ashikaga, Takashi Noda, Kazuhiro Satomi, Hiroshi Tada, Masahiko Takagi, Satoshi Yasuda, Kengo Kusano

**Affiliations:** ^1^ Department of Cardiovascular Medicine National Cerebral and Cardiovascular Center Suita Japan; ^2^ Department of Cardiovascular Medicine University of Fukui Yoshida Japan; ^3^ Department of Medicine II Kansai Medical University Hirakata Japan; ^4^ Second Department of Internal Medicine University of Toyama Toyama Japan; ^5^ Section of Arrhythmia Division of Cardiovascular Medicine Department of Internal Medicine Kobe University Graduate School of Medicine Kobe Japan; ^6^ Department of Cardiovascular Medicine Miyazaki Medical Association Hospital Miyazaki Japan; ^7^ Department of Cardiovascular Medicine Tokyo Medical University Hospital Tokyo Japan

**Keywords:** AF, cryoballoon, RF ablation

## Abstract

**Background:**

Catheter ablation of atrial fibrillation (AF) is increasingly performed worldwide in patients with heart failure (HF). However, it has been recently emphasized that AF ablation in patients with HF is associated with increased risks of procedure‐related complications and mortality. There are little data about the differences in the efficacy and safety between cryoballoon (CB) and radiofrequency (RF) ablation of AF in patients with HF.

**Methods:**

The CRABL‐HF study is designed as a prospective, multicenter, open‐label, controlled, and randomized clinical trial comparing the efficacy and safety of AF ablation between CB and RF ablation in patients with HF (LVEF ≤40%) (UMIN Clinical Trials Registry UMIN000032433). The CRABL‐HF study will consist of 110 patients at multicenter in Japan. The patients will be registered and randomly assigned to either the CB ablation or RF ablation group with a 1:1 allocation. The primary endpoint of this study is the occurrence of atrial tachyarrhythmias (ATs) at 1 year with a blanking period of 90 days after ablation. Key secondary endpoints are the success rate of the pulmonary vein isolation, total procedural time, left atrial dwelling time, total fluoroscopy time, radiation exposure, complication rate, composite of all‐cause mortality or HF hospitalizations, cardiovascular events, change in left ventricular ejection fraction, and change in quality of life.

**Results:**

The results of this study are currently under investigation.

**Conclusion:**

The CRABL‐HF study is being conducted to compare the efficacy and safety of catheter ablation of AF between CB and RF ablation in patients with HF.

## Graphical abstract

There are little data about the differences in the efficacy and safety between cryoballoon (CB) and radiofrequency (RF) ablation of atrial fibrillation (AF) in patients with heart failure (HF). The CRABL‐HF study is being conducted to compare the efficacy and safety of catheter ablation of PAF between CB and RF ablation in patients with HF.

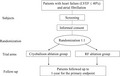

## 1 INTRODUCTION

Atrial fibrillation (AF) often coexists with heart failure (HF) and the presence of AF in patients with HF is associated with increased risks of the hospitalizations, strokes, and mortality.^1^, ^2^ Electrical pulmonary vein isolation (PVI) by catheter ablation has been established as an essential treatment for AF since Haissaguerre et al first reported the presence of arrhythmogenic triggers in the muscular sleeves of pulmonary veins (PV).^3^ In patients with both HF and AF, catheter ablation has been shown to be superior to all other strategies such as medical therapy or cardiac implantable electronic devices plus an atrioventricular node ablation with significant improvements in the mortality, HF hospitalizations, left ventricular ejection fraction (LVEF), NYHA functional classification, 6 minute walk test, and quality of life (QoL).^4^, ^5^, ^6^, ^7^

The CASTLE‐AF study was a multicenter, randomized trial, which assessed the outcome of radiofrequency (RF) ablation of AF compared with medical therapy in patients with HF.^8^ The study proved that RF ablation of AF in patients with HF reduced the all‐cause mortality and HF hospitalizations as compared to medical therapy. Although the most widely adopted and established technique for AF ablation is RF ablation, which was also used in the CASTLE AF, the technical complexity of RF ablation generally demands a long learning curve and relatively long procedure time.^9^ Recently, the cryoballoon (CB; Arctic Front Advance; Medtronic) technology has emerged to simplify the PVI.^10^, ^11^ The FIRE and ICE study was a multicenter, randomized, noninferiority trial to compare the two different technologies, CB and RF ablation, in patients with paroxysmal AF. CB ablation is noninferiority to RF ablation with respect to the efficacy and safety.^12^ Furthermore, the total procedure time, left atrial (LA) dwelling time, and total fluoroscopy time were significantly shorter in CB ablation than RF ablation. It is noted that patients with a reduced LV contraction were excluded from the FIRE and ICE study.

Recently, AF ablation has been increasingly performed in patients with HF.^13^, ^14^ However, the underlying pathophysiology differs between patients with and without HF.^15^, ^16^ Further, the fundamental mechanism of catheter ablation differs between CB and RF ablation as well. There have been no randomized controlled trials addressing the differences in the clinical outcomes between CB and RF ablation for the treatment of AF in patients with HF. This study will aim to prospectively compare the efficacy and safety of catheter ablation of AF between CB and RF ablation in patients with HF.

## 2 METHODS

### 2.1 Aim

The aim of this study is to compare the efficacy and safety of catheter ablation of AF between CB and RF ablation in patients with HF.

### 2.2 Study design

**Figure 1:**
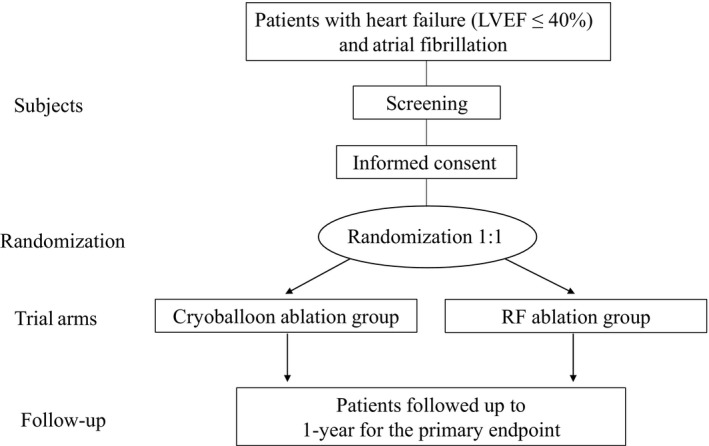
Flow chart of the study. LVEF, left ventricular ejection fraction

The CRABL‐HF study is designed as a prospective, multicenter, open‐label, controlled, and randomized, noninferiority clinical trial comparing the efficacy and safety of AF ablation at 1 year with a blanking period of 90 days after ablation between CB and RF ablation in patients with HF from 25 April 2018 to 31 March 2027 (Figure 1). The enrollment period is from 25 April 2018 to 31 March 2023. Eligible subjects will be randomly assigned to either the CB ablation or RF ablation group (allocation rate 1:1). The randomization will be stratified by the LA diameter. The study period will be extended if the number of enrollments is not be achieved during the period.

### 2.3 Patient eligibility and recruitment

Male and Female patients aged 20‐85 years with both HF and AF, and scheduled for catheter ablation of AF are eligible for the study. It is necessary for eligible subjects to be diagnoses with AF with at least one episode documented (≥30 seconds) and to have an LVEF ≤40%. Patients will be excluded if they have histories of previous catheter ablation and/or surgical procedure of AF before providing informed consent, or LA dimeter >55 mm (parasternal long‐axis view).

### 2.4 Study procedures

#### 2.4.1 The CB ablation

The detailed procedure has been described elsewhere.^11^ The fourth‐ or second‐generation CBs will be used in the CB ablation group. In brief, an atrial transseptal puncture will be performed using a standard Brockenbrough technique. The 28 mm CB will be introduced into the LA through a steerable sheath (FlexCath Advance, Medtronic) and be inflated proximal to each PV and pushed gently, aiming for complete sealing at the antral aspect of the PV. Contrast medium will be injected from the distal lumen of the CB to confirm the exact position of the inflated balloon in relation to the PV ostium. A 180 second freeze cycle will be performed at each PV.^11^ When the initial freezing fails to isolate the PV, the CB will be repositioned and a second freezing cycle will be applied. No further freezing cycle will be applied when PVI cannot be achieved after a maximum of five freezing cycles per vein. When the CB ablation cannot achieve the PVI, the steerable sheath (FlexCath Advance) will be exchanged to another sheath (SL0 or Agilis, Abbott) and additional focal ablation will be performed with Freezor Max (Medtronic) or RF catheter.

#### 2.4.2 RF ablation

The detailed procedure has been described elsewhere.^17^ The three‐dimensional (3D) geometry of the LA and PVs will be depicted by a circular mapping catheter or PentaRay (Biosense Webster) by the use of a 3D mapping system; CARTO (Biosense Webster), NavX (Abbott), or Rhythmia (Boston Scientific). The ablation of the ipsilateral superior and inferior PVs will be jointly performed under navigation using the 3D mapping system. RF ablation will be performed with an irrigated 3.5 or 8 mm tip electrode catheter (Thermocool SmartTouch; Biosense Webster, TactiCath; Abbott, or Intella Tip MiFi; Boston Scientific). The RF ablation settings will be according to the center's standard of care, generally with a power of 30‐40 W, targeting an ablation index of 450‐550 for CARTO and lesion index of 4.0‐5.0 for NavX. The power and duration will usually be reduced to 20‐25 W for 20 seconds on the LA posterior wall near the esophagus.

### 2.5 Follow‐up

Follow‐up will be conducted at 1 month, and then every 1‐3 months following the ablation procedure with clinic visits. Patients will be scheduled examinations, including 12‐lead ECGs, 24 hour Holter recordings (3, 12, 24, and 36 months after the procedure), and/or ambulatory electrogram recorder (HCG‐801, OMRON Healthcare Co., Ltd). In patients with cardiac implantable electronic device, home monitoring will be adapted to facilitate the continuous monitoring of AF episodes. In patients without cardiac implantable electronic device, one‐channel electrocardiograms will be recorded twice daily for 1 year after the procedure except for the blanking period of the 90 days with the use of ambulatory electrogram recorder. Echocardiography and QoL questionnaires will also be obtained during the follow‐up (3, 12, 24, and 36 months after the procedure). LVEF will be recommended to be calculated during sinus rhythm. The QoL will be assessed by AFEQT questionnaires.^18^ All documented atrial tachyarrhythmias (ATs) lasting ≥30 seconds occurring outside the blanking period of 90 days will be considered as a recurrence. In the case of recurrences outside the after ablation blanking period, it will be recommended to perform a second catheter ablation. The sum of the durations of all episodes of ATs regardless of lasting ≥30 seconds or not, will be calculated and expressed as a percentage with respect to 24 hours to assess the AF burden in patients with cardiac implantable electronic device.

Adverse events (AE) will be collected during the study periods. An AE is defined as any untoward medical occurrence in a subject in this study, regardless of whether the causal relationship of the AE with the ablation procedure.

### 2.6 Inclusion and exclusion criteria

#### 2.6.1 Inclusion criteria

Subjects must meet all of the following criteria:

1. Diagnosed with HF with an LVEF ≤40%
2. Diagnosed with AF with at least 1 episode documented (≥30 seconds) in accordance with the 2014 AHA/ACC/HRS guidelines^17^
3. Age 20‐85 years
4. Capable of complying with the protocol and providing written informed consent.

#### 2.6.2 Exclusion criteria

Those who meet any of the following criteria are ineligible for the study:

1. Previous catheter ablation and/or surgical procedure of AF
2. LA diameter >55 mm (parasternal long‐axis view)
3. Woman currently or possibly pregnant
4. Enrollment in another investigational drug and/or device study.

### 2.7 Outcomes

#### 2.7.1 Primary endpoint

The primary endpoint of this study is the occurrence of ATs at 1 year with a blanking period of 90 days after ablation.

#### 2.7.2 Secondary endpoints

The key secondary endpoints are as follows:

1. The success rate of the PVI
2. Total procedural time
3. LA dwelling time
4. Total fluoroscopy time
5. Radiation exposure
6. Complication rate
7. Occurrence of ATs at 3 years after ablation
8. Composite of all‐cause mortality or HF hospitalizations at 1 and 3 years after ablation
9. All‐cause mortality at 1 and 3 years after ablation
10. HF‐hospitalizations at 1 and 3 years after ablation
11. Cardiovascular mortality at 1 and 3 years after ablation
12. Cardiovascular events at 1 and 3 years after ablation
13. Change and percentage change in LVEF at 1 and 3 years after ablation
14. Change and percentage change in QoL at 1 and 3 years after ablation
15. Occurrence of ATs at 1 and 3 years after the latest ablation

### 2.8 Other variables

1. Change and percentage change in brain natriuretic peptide at 1 and 3 years after ablation
2. Change in NYHA functional class at 1 and 3 years after ablation
3. Change in LV diastolic/systolic diameter, LA diameter, tissue doppler index E/e' at 1 and 3 years after ablation
4. Change and percentage change in AF burden at 1 and 3 years after ablation
5. Change and percentage change in serum creatinine, estimated creatinine clearance, estimated glomerular filtration rate at 1 and 3 years after ablation
6. Change and percentage change in LA mean pressure before and after procedure
7. Perioperative volume of infusion and urine
8. Interaction between ablation outcome and LVEF, LV diastolic/systolic diameter, LA diameter, tissue doppler index E/e', serum creatinine, estimated creatinine clearance, estimated glomerular filtration rate, LA mean pressure before and after procedure, and patient characteristics at baseline

### 2.9 Sample size

The estimated sample size is 110 (55 in each group). The sample size was calculated based on the primary hypothesis. The recurrence rate of ATs after an ablation of AF has been reported to be comparable between the CB and RF ablation in patients without HF, ranging 65%‐90%,^11^, ^12^, ^19^, ^20^ and the recurrence rate of ATs after CB ablation in patients with HF with an LVEF ≤40% has been reported to be equal to those without HF.^21^ Therefore, we hypothesized that the incidence of recurrent ATs after an ablation procedure could be 20% in the AF patients from previous studies in both the CB and RF ablation groups. The sample size for the randomized comparison was calculated as 51 patients per group with a power of 80% by a two‐sided 95% confidence interval for the difference in the population proportions with a width that is equal to 0.35 when the two estimated group sample proportions are equally 0.20. To cope with a potential loss‐to‐follow‐up, a minimum of 55 patients per group will be enrolled in the study. Withdrawn patients will not be replaced.

### 2.10 Randomization and allocation factor

The CRABL‐HF will consist of 110 patients at multicenter in Japan. The patients will be registered and randomly assigned to either the CB ablation or RF ablation group with a 1:1 allocation. We will use the minimization method proposed by Pocock and Simon to allocate the patients with an in‐house validated mail‐based system. Randomization will be stratified by the LA diameter, which may influence the evaluation of the efficacy of the ablation procedure,^22^ providing a balanced treatment assignment in both cohorts (<50 vs ≥50 mm). The patients' group allocations will be revealed to the operator at least 1 day before the ablation procedure. The information regarding the number of allocated subjects in each group will not be disclosed to the principal investigator or investigator until the completion of the study.

### 2.11 Data quality control and management

The principal investigator will authorize access to the electronic Case Report Form (CRF) system for investigators. The principal investigator will take full responsibility for the accuracy and reliability of all the data entered in the CRFs. The principal investigator and other investigators must not disclose the information contained in the CRFs to third parties. Only investigators can access data.

### 2.12 Statistical analysis

Comparisons between the randomized groups will be performed on the basis of the intention‐to‐treat (ITT) principle. A per‐protocol basis analysis will also be performed to assess the robustness of the conclusions derived from the ITT basis analysis. The Kaplan‐Meier method will be used to estimate the survival curve, and the log‐rank test will be used to compare the curves between the groups for the time‐to‐event variables. Categorical variables will be analyzed using a chi‐square test or Fisher's exact test. Continuous variables will be presented as the mean ± standard deviation or median with the interquartile range (25th‐75th percentiles) and compared with the Student's t test or Wilcoxon rank‐sum test, as appropriate. A *P* value < .05 will be considered significant. Up‐to‐date versions of JMP (SAS institute) will be used to conduct analyses. The patient demographic data and outcome of catheter ablation in each group will be collected descriptively as presented in Tables 1 and 2.

**Table 1:** Patient characteristics at baseline

Age, years, n (%)
Male sex, n (%)
Height, cm
Weight, kg
NYHA functional class
History of a heart failure hospitalization
Number of heart failure hospitalizations
Blood pressure, mm Hg
Heart rate, /min
Duration of atrial fibrillation, months
Congestive heart failure, n (%)
Hypertension, n (%)
Diabetes mellitus, n (%)
Stroke and/or transient ischemic attack, n (%)
Structural heart disease, n (%)
Coronary artery disease
Valvular heat disease
Dilated cardiomyopathy
Hypertrophic cardiomyopathy
Others
Postopen heart surgery
CHADS_2_ score
CHA_2_DS_2_‐VASc score
Echocardiographic data
Left ventricular ejection fraction, %
Left atrial dimension, mm (%)
Left atrial dimension, <50 mm
Left atrial dimension, ≥50 mm
Computed tomography data
Pulmonary vein diameter, mm
Cardiac implantable electronic device
Pacemaker
ICD
CRT‐P
CRT‐D
History of anti‐arrhythmic drug use, n (%)
Disopyramide, n (%)
Cibenzoline, n (%)
Aprindine, n (%)
Pilsicainide, n (%)
Flecainide, n (%)
Propafenone, n (%)
Bepridil, n (%)
Sotalol, n (%)
Amiodarone, n (%)
Verapamil, n (%)
Beta‐blocker, n (%)
Digitalis, n (%)
Others, n (%)
Laboratory data

**Table 2:** The ablation procedure and complications

Procedure time (groin puncture to catheter extraction), min
Left atrial dwelling time
Fluoroscopic time, min
For cryoablation
Total freezing cycles, n
Total freezing time, s
The need for touch up RF ablation, n
For RF ablation
Total RF application time, s
Other adjunctive ablation, n (%)
Use of 3D mapping system, n (%)
Complications, n (%)
Pericardial effusion requiring drainage
Pericardial effusion not requiring drainage
Transient ischemic attack
Cerebral infarction
Other thromboembolisms
Transient phrenic nerve paralysis
Prolonged phrenic nerve paralysis
Severe pulmonary vein stenosis
Hematoma at the puncture site
Pseudoaneurysm at the puncture site
Gastric hypomotility
Others
Death
Discharge prescription, n (%)
Oral anticoagulant
Vitamin‐K antagonist
Direct oral anticoagulant
Antiarrhythmic drugs
Disopyramide
Cibenzoline
Aprindine
Pilsicainide
Flecainide
Propafenone
Bepridil
Sotalol
Amiodarone
Verapamil
Beta‐blocker
Digitalis
Others
Angiotensin converting enzyme inhibitor
Angiotensin II receptor blocker
Angiotensin receptor‐neprilysin inhibitor
Mineralocorticoid Receptor Antagonists
Ivabradine
Loop diuretic
Statin

Abbreviation: RF, radiofrequency.

### 2.13 Study organization

The research group consists of investigators at multicenter in Japan and an independent data monitoring committee. All study investigators will have completed at least 50 procedures with each study technique (CB ablation and RF ablation) to be able to participate in this study.

### 2.14 Ethics

The study is registered at the UMIN Clinical Trials Registry (UMIN Clinical Trials Registry UMIN000032433). The study is being conducted in accordance with the Declaration of Helsinki and the Ethical Guidelines for Clinical Studies issued by the Ministry of Health, Labour and Welfare, Japan. This study received approval from the institutional review board (IRB) of the National Cerebral and Cardiovascular Center, Japan (M29‐174, April 25, 2018), along with the IRBs of all participating institutions. All participants will provide written informed consent.

## 3 RESULTS

The results of this study are currently under investigation.

## 4 DISCUSSION

There are some debatable questions about catheter ablation of AF in patients with HF. These questions will be addressed by the CRABL‐HF study.

### 4.1 Impact of the difference in the PVI area and durability between CB and RF ablation on the efficacy

Patients with HF have elevated filling pressures and larger PV ostia, and this means that a larger isolation area may be required in patients with both HF and AF.^23^, ^24^ Some studies have reported the efficacy of catheter ablation of AF in patients with HF with respect to the mortality, HF hospitalization, and LVEF; however, it is noted that the ablation strategies in almost all of those studies allowed for a substrate based ablation such as a posterior isolation in addition to the PVI.^5^, ^6^, ^7^, ^8^ Therefore, it is possible that a wider area of the PVI may increase the efficacy of the catheter ablation of AF in patients with HF, and the PVI area is reported to be wider in RF ablation than CB ablation.^25^

The PV antrum for the PVI in patients with HF may be more thickened compared to that in those without HF because patients with HF have atrial hypertrophy.^15^, ^16^ The transmural lesion creation by the PVI may be difficult in patients with HF and the importance of the PVI durability may be emphasized more in those patients. Regarding the PVI durability, the CB ablation has some advantages as compared to RF ablation.^26^, ^27^

### 4.2 Impact of the difference in the procedure process and lesion creation between CB and RF ablation on the safety

It is notable that, despite advances in the catheter ablation technologies and increased operator experience year by year, AF ablation procedure‐related complications and mortality have been rather increasing.^13^, ^14^ The presence of HF is associated with increases in the mortality and procedural complications.^13^, ^14^ Therefore, we still should pursue a better technology for catheter ablation of AF in patients with HF even though catheter ablation of AF in patients with HF has been thought to be highly safe based on studies from highly experienced centers.^4^, ^5^, ^6^, ^7^, ^8^ Regarding the safety, Chun et al found that the risk of cardiac tamponade is lower in CB ablation of AF than RF ablation, although the total complication risk including phrenic nerve palsy is higher in CB ablation.

## 5 CONCLUSIONS

The CRABL‐HF study will be conducted to compare the efficacy and safety of catheter ablation of AF between CB and RF ablation in patients with HF.

## CONFLICT OF INTEREST

Authors declare no conflict of interests for this article.
